# Devices for Prevention of Atrial Tachyarrhythmias

**Published:** 2004-04-01

**Authors:** Ignacio Fernández Lozano, Jorge Toquero, José Antonio Fernández Diaz, Bogdan Ionescu, Vanesa Moñivas, Pilar Ortiz, Beatriz Fuertes, Luis Alonso Pulpón

**Affiliations:** Clinica Puerta de Hierro, Madrid

## Introduction

Atrial fibrillation (AF) is the most frequent sustained cardiac arrhythmia in clinical practice and, although its importance has been underestimated even in recent years, we are now becoming aware of its clinical transcendence [1-3]. The classical treatment is pharmacological, but its efficacy is limited and it does have side effects [4-5]. Therefore, in recent years, there has been an increasing interest in other types of non-pharmacological treatments [6,7].

Physiologic cardiac pacing has proven to be more effective than VVI mode pacing to prevent the occurrence of AF during the follow-up of patients who have had a permanent pacemaker implanted [8-10]. There are currently different lines of research that use different atrial pacing techniques to prevent and treat episodes of paroxysmal atrial fibrillation [11,12]. Techniques of multi-site pacing in the right atrium or both atria, new atrial pacing sites, prevention algorithms for paroxysmal atrial fibrillation episodes, and even high-frequency atrial tachyarrhythmia termination algorithms have all been proposed. In this article, we will try to synthesize the grounds for and findings of the different lines of research currently being developed.

## Pacing Activation Mechanisms

The occurrence of atrial fibrillation in a patient depends on the interaction of three factors: substratum, triggers and modulating factors. The substratum is the usually sick atrial myocardium with areas of fibrosis that result in heterogeneous refractoriness that facilitates the appearance of functional reentry areas. Shortening the refractory periods that take place in atrial myocardium as a result of electrical remodeling favors the perpetuation of AF [13]. The triggers are the atrial extra-systoles or episodes of atrial tachycardia or flutter that in many cases precede episodes of AF [14]. The modulating factors such as sympathicotonia or circulating catecholamines facilitate the occurrence and sustainability of AF.

Atrial pacing can prevent the development of AF through several mechanisms. It can prevent dispersion of the atrial refractory periods associated with bradycardia. This effect can be particularly beneficial in cases of vagotonic AF [15,16]. New approaches of interatrial septal, Bachmann or simultaneous dual-site pacing have demonstrated an enhanced speed of conduction of the electric pulse through the atrium. This higher speed of conduction is reflected in a shorter duration of the P wave of the superficial electrocardiogram [17-19] and can help to prevent AF.

Atrial pacing at frequencies higher than the basal frequency can prevent the occurrence of AE by overdrive suppression, or at least significantly decrease their number. It can also decrease the post-AE pauses that result in high dispersion in the refractory periods. In addition, it can suppress the increased automatism that is responsible for focal AF [20].

The new techniques of multi-site pacing can improve the hemodynamics of sick atria, and this hemodynamic improvement can in itself prevent episodes of  AF [21]. Finally, the episodes of high-frequency atrial tachycardia that often precede the occurrence of AF can, on occasions, be terminated with atrial pacing [22].

## Patients with Permanent Pacing Indication

In 1994, Andersen and Cols [23] published the results of the first randomized trial that compared physiologic pacing with VVI mode. It included 225 patients with sinus node dysfunction and narrow QRS and an average age of 76 years who were randomized to receive an AAI versus VVI pacemaker. The long-term follow-up of these patients was published in 1997 [8]; the outcome, after 5.5 years of follow-up, was a lower rate of AF, a lower cardiovascular mortality and a significant reduction in overall mortality in patients randomized to the AAI modality. Subsequent publications of this same population showed a lower number of cases of progression to heart failure, improved echocardiographic parameters [24] and fewer thromboembolism events [25].

In 1998, the results of the PASE (“Pacemaker Selection in the Elderly”) study were published [26]. This study randomized 407 patients over 65 years of age and with bradycardia to receive a DDDR versus VVIR pacemaker. After an average follow-up of 2.5 years, the quality of life endpoint significantly improved in both groups (p<0.001) compared to the baseline situation, but no significant differences were detected between the two groups except for a slight difference in favor of using the DDDR pacemaker in patients with SND. No differences were found in number of deaths, ACVA or admissions for heart failure. However, a lower incidence of AF in the DDDR randomized group was demonstrated. The results of the study were partially clouded because a very high rate of crossover (26%) from the VVIR to DDDR group was recorded due to pacemaker syndrome during follow-up. This may have undervalued the benefit of DDDR pacing in this type of patient.

That same year, Mattioli published the results of a prospective study that analyzed the incidence of atrial fibrillation as a function of the pacing modality [27]. It included 210 patients, 110 with SND and 100 with AV block, who received a physiologic PM (AAI, DDD, DDDR or VDD) versus a ventricular PM (VVI or VVIR). Patients with a background of atrial fibrillation were excluded, and the incidence of AF was 10% during the first year, 23% after 3 years and 31% after 5 years. The patients randomized to a physiologic PM had a lower risk of developing AF during follow-up, and the greatest benefits were for patients with SND.

The CTOPP (Canadian Trial of Physiologic Pacing) [28] randomized 2568 patients with pacemaker indication, 1474 to ventricular pacing (VVI or VVIR) and 1094 to physiologic pacing (AAI, AAIR, DDD or DDDR). After an average follow-up of 3 years, no significant differences were found in the combined endpoint of cardiovascular mortality and ACVA between both groups. However, the annual incidence of atrial fibrillation was 5.3% in the physiologic pacing group versus 6.6% in the ventricular pacing group. A relative risk reduction of 18% was statistically significant (p=0.05). A subsequent analysis of this same study published in 2001 [29] demonstrated that physiologic pacing was very beneficial for the patients most dependent on pacemakers. Thus, those patients with an intrinsic cardiac frequency of less than 60 bpm during the first follow-up had a lower rate of cardiovascular death or ACVA and a lower total mortality (p<0.001).

The MOST trial, published in 2002, randomizes 2010 patients with SND to receive a VVIR versus DDDR pacemaker [30]. After completing an average follow-up of 2.7 years, the primary endpoint of ACVA or total mortality occurred in 22.2% of the patients, and no significant differences were observed between the two groups (p=0.32). However, a lower incidence of AF was observed in the DDDR randomized group (p=0.008). The occurrence of permanent AF was 26.7% in the VVIR group versus 15.2% in the DDDR group (p=0.001).

The preliminary findings of the UK-PACE trial [31] were reported during the 2003 Congress of the American College of Cardiology. This trial randomizes 2000 patients with AV block to a DDDR versus VVIR pacemaker, and no differences are found in total or cardiac mortality between the two groups.

In all, almost 7000 patients have been included in randomized trials, and there is indisputable proof of a lower incidence of atrial fibrillation and less progression to chronic AF when a physiologic pacemaker is used in patients with permanent pacing indication.

##  Patients Without Associated Bradycardia

In 1999, the results of PA^3^ [32] were published. This study assesses the efficacy of atrial pacing in patients with paroxysmal atrial fibrillation and without permanent pacing indication. In order to be included, the patients had to have had at least three episodes of paroxysmal AF during the preceding year in spite of having received correct antiarrhythmic treatment. A dual-chamber pacemaker was implanted in 97 patients, and they were randomized to DDIR programming at 70 bpm or DDI at 30 bpm. The primary endpoint was the time up to the first recurrence of an atrial tachycardia lasting more than 5 minutes. The counters of the device, which did not have intracavity electrographs, were used for this purpose. This endpoint did not differ significantly in the two groups.

Subsequently, AV node ablation was performed in 67 patients, randomizing the DDDR PM at 70 bpm minimum frequency or VDD PM at 60 bpm [33]. There were also no differences detected in the recurrence of AF in this second phase.

This study has some methodological limitations, the main one being a high rate - 25% - of group crossover from the DDI mode at 30 bpm to the DDIR mode at 70 bpm. In addition, the DDIR programming only achieved 67% atrial pacing, and other studies have demonstrated that the higher the percentage of pacing, the greater is the efficacy in preventing AF. Finally, the primary endpoint is based on analysis of the PM counters, without electrographs that confirm the recurrence of the arrhythmia.

In spite of all this, an analysis of the results of PA^3^  puts in doubt the efficacy of atrial pacing in preventing episodes of AF in patients with associated bradycardia.

## Pacing at Special Sites

### A) Biatrial Pacing

The association between disorders of intra- and inter-atrial conduction and the occurrence of AF episodes has been known for some years [34]. Techniques of simultaneous pacing in both atria [35] have been proposed to correct this. This technique uses an electrode located inside the coronary sinus and another in the right atrium; both are jointly connected to the atrial port by a Y-connector. Some second-generation PMs have algorithms that resynchronize both atria in case an event sensed in the opposite atrium occurs [36].

The French group directed by Dr. Daubert publishes the experience of a single center over a period of 9 years [37]. It demonstrates a reduction of the P wave from 187 to 106 msec with biatrial pacing. After an average follow-up of 34 months, a third of the patients is free of arrhythmias, a third presents paroxysmal episodes but with maintenance of sinus rhythm, and the remaining third has permanent atrial fibrillation.

This technique has also been effectively used to prevent episodes of AF after heart surgery [38-40]. However, the technique is limited by the need for 2 electrodes, the high rate of displacement of the coronary sinus electrode and the use of Y-connectors.

### B) Dual-Site Right Atrial Pacing

In this technique, an electrode is implanted in the right appendage and another in the coronary sinus ostium. This succeeds in reducing the conduction time and achieves a more homogeneous atrial activation, thus eliminating the displacement problems of electrodes placed inside the coronary sinus.

Early publications [19] demonstrated a beneficial effect of dual-site pacing versus single-site pacing or no pacing. In a prospective randomized study that includes 118 patients, dual-site pacing only demonstrates a tendency for longer times without arrhythmias. In the subgroup of patients that continues under antiarrhythmic treatment, dual-site pacing from the right atrium significantly reduces the number of atrial arrhythmia episodes.

### C) Pacing at Special Sites

The atrial pacing site is of utmost importance to the total conduction time. To try to achieve the potential beneficial effects of dual-site pacing, different pacing sites in the right atrium have been proposed, specifically Bachmann's bundle [18] and the coronary sinus ostium [42]. In a randomized study, Padeletti analyzes the effects of pacing in 46 patients with a history of AF. During the pacing phase in the right appendage, the number of symptomatic AF episodes was reduced from 6 to 2 a month, whereas with interatrial septum pacing the number was reduced from 5 to 0.2 a month. In another study [44], septal pacing resulted in a subjective 68% improvement of symptomatic episodes and an objective 60% reduction of the incidence of AF.

The results with Bachmann's bundle pacing are promising [45]. The electrode is easily positioned by using a simple fluoroscopic reference, and this position is associated with a marked reduction in the P wave duration. In a prospective study that included 120 patients, pacing from Bachmann's bundle reduced the incidence of progression to permanent AF (45 versus 75%, p<0.05) after an average follow-up of one year.

In short, both techniques have proved to be somewhat useful, although in trials with short follow-up periods and with a small number of patients. Of the two positions, Bachmann's bundle offers the advantage of causing fewer problems in ventricular signal detection. The far field can be significant in this group of patients; it limits the utility of mode exchange algorithms and introduces incorrect information in the pacemaker event counters.

## Prevention Algorithms

**Figure 1:**
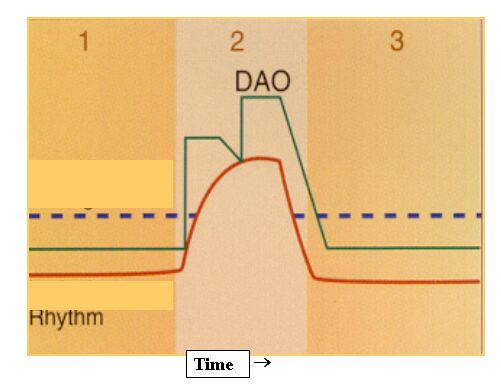
Example of atrial overdrive pacing algorithms. DAO continuously monitors the atrial channel for intrinsic atrial events. When two P-waves are sensed within 16 cardiac cycles, the atrial pacing rate is increased. The P waves do not need to be consecutive. Following the delivery of the programmed number of DAO pacing cycles, pacing cycle length is extended or increased to facilitate the search for intrinsic atrial activity. Once atrial activity is sensed and meets the above criteria, the algorithm will automatically calculate and provide an incremental A pacing rate just above the patients own circadian rhythm.

Different atrial pacing algorithms have been developed, all designed for atrial overdrive pacing and prevention of AF episodes. Basically they can be divided into four types [46]: atrial overdrive pacing algorithms (Figure 1), response to atrial extrasystoles (to prevent short-long sequences), response to sinus rhythm recovery, and post-exercise relative bradycardia sequence prevention algorithms. The most commonly used algorithm is intended to stimulate the atrium just above the sinus frequency to achieve unified atrial refractory periods. The higher the atrial pacing percentage, the greater the effectiveness. However, excessively high frequencies are perceived by the patient as a disagreeable sensation. Therefore, the objective is to achieve a high percentage of atrial pacing without excessively increasing the patient's frequency.

The efficacy of an atrial overdrive pacing algorithm has been analyzed in the ADOPT-A trial [47]. A total of 288 patients with sinus node dysfunction and a background of paroxysmal or persistent AF received a DDDR pacemaker with a lower frequency programmed at 70 bpm. In a parallel design, the patients were randomized to activate or not activate the overdrive pacing algorithm. After a 6-month follow-up, it was confirmed that activation of the algorithm caused an increase in the pacing percentage of 92.9% versus 67.9% (p<0.001). The atrial fibrillation percentage dropped 25% with the algorithm activated (2.5% vs 1.87%, p=0.005). The number of rehospitalizations or the need for cardioversion did not differ between the two groups.

The AFT (AF Therapy) Trial included 372 patients with paroxysmal AF, with or without permanent pacing indication, in a complex protocol divided into 4 phases [48]. The first phase of the study was a monitoring phase intended to obtain information on the way AF begins in this population. The second phase compares the efficacy of conventional pacing. During the third phase of the study, the efficacy of four combined pacing algorithms is analyzed and compared to conventional DDDR pacing at 70 bpm. Unfortunately, it has only been possible to analyze data from 97 patients because information was lost during follow-up and because of detection of ventricular activity in the atrial channel. The prevention algorithms achieved a 34% reduction of the AF rate (p<0.05). A subsequent subanalysis of this study [49] showed how conventional pacing was very effective in patients with AF and bradycardia, whereas the algorithms tended to be more useful for those patients with prior AF and without bradycardia.

The ASPECT trial (Atrial Septal Pacing Efficacy Clinical Trial) included 298 patients who were randomized to receive the atrial electrode in the septum or another position in the right atrium. After one month, those patients with recurrence of AF were randomized to conventional pacing or algorithms activated during a 3-month phase. At the end of this phase, the PM of the opposite group was reprogrammed. The PM counters did not show evidence of any significant differences with activation of the algorithms, nor were any objective differences based on the electrode position found, except that the symptomatic episodes were less frequent in those patients who had an electrode implanted in the interatrial septum.

## PREVENT Register

Atrial fibrillation is a complex arrhythmia. Phase 1 of the AFT study [48] demonstrated that there are multiple mechanisms of AF initiation that differ in different patients. Nowadays, modern PMs have numerous diagnostic functions that help to characterize the way AF begins in a specific patient. In addition, the prevention algorithms generally vary, and it may be that not all of them are beneficial in all patients. The prospective studies have a strict protocol that makes it difficult to optimize programming for a specific patient. Therefore, to study the efficacy of the AF prevention algorithms in the real world by using all the advantages of a PM in a particular patient, we have designed the Prevent Register [51]

The Prevent-AF Register is prospective, non-randomized and multicentric. It includes candidate patients for permanent pacing due to sinus node dysfunction (type I or IIa indication of the AHA-ACC) [52] with or without prior paroxysmal atrial fibrillation.

Four preventive pacing algorithms are incorporated: Pace Conditioning™, Post-PAC Response™, PAC Suppression™, Post-Exercise Response™. The Pace Conditioning detects the patient's basal frequency and increases the pacemaker pacing frequency to increase the percentage of paced beats, in an attempt to maintain patients slightly above their basal frequency. The PAC Suppression is activated if atrial extrasystoles are detected, increasing the basal pacing frequency in an attempt to suppress them. The Post-PAC Response prevents the compensatory pause after an atrial extrasystole by eliminating the short-long sequences. The Post-Exercise Rate Control prevents abrupt frequency drops after episodes of physical exercise. The Register was implemented in 14 centers in Spain. A total of 68 patients (33 men, 35 women) were included from April 2000 to April 2001. The average age was 72±12 years and the pacing indication was sinus node dysfunction in all cases. A total of 53 patients (78%) had had documented paroxysmal AF episodes prior to implant.

The recurrence of at least one episode of paroxysmal atrial fibrillation was documented in a total of 32 patients during the monitoring phase, and at least one subsequent phase of preventive pacing was completed. The preventive algorithms were programmed on an individualized basis, based on information from the AF episode commencement mode.

**Figure 2:**
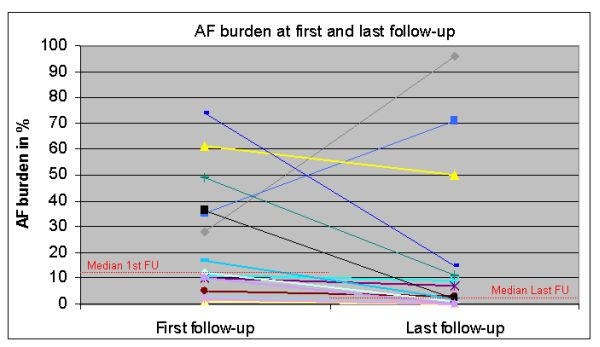
Change in AF Burden during follow up. Median AF burden reduced from 12% to 3%.

The average atrial arrhythmic burden was reduced in the total group (32 patients) from 0.94 to 0.3 hours a day (Wilcoxon test: p=0.034) (Figure 2). In relative values, the average atrial arrhythmic burden dropped from 3.9% to 1.3%, i.e. a 67% reduction. The mean atrial pacing percentage rose from 72% to 78%, i.e. an increase of only 13%.

The Register results confirm the usefulness of latest-generation pacemakers both for diagnosing episodes of paroxysmal atrial fibrillation and for preventing them by means of pacing algorithms. The diagnostic functions of the pacemaker provided relevant information on the occurrence and development of atrial fibrillation in each patient. In this way, the most suitable algorithms for preventing AF were programmed on an individualized basis.

## Atrial Tachycardia Termination Algorithms

**Figure 3:**
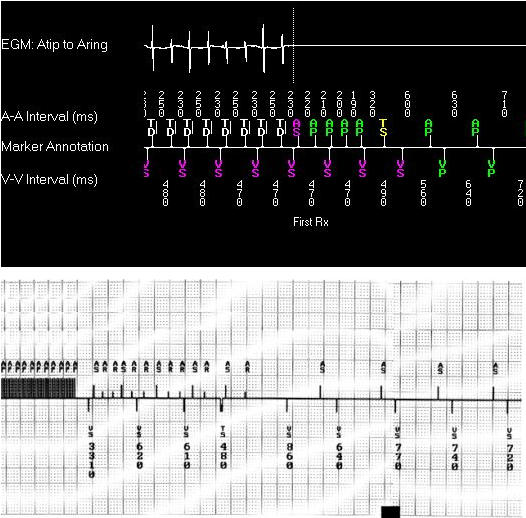
Overdrive pacing algorithms.

Many episodes of AF begin preceded by a rapid, relatively regular atrial tachycardia. Some of these atrial tachycardias can be eliminated by overdrive pacing in a way similar to overdrive pacing and termination of other tachycardias by reentry. To end atrial tachycardias, algorithms similar to those used in implantable defribillators have been employed, and specific algorithms have been designed to act in the atrium as 50 Hz bursts (Figure 3).

These therapies have been tested in 537 patients possessing a DAI with atrial therapies [53]. After an average follow-up of 11.4 months, the overall efficacy of the algorithms was 48%, with 59% in cases of atrial tachycardia and 30% in those cases classified as AF. The efficacy depends on the arrhythmia cycle length, and it is lower the faster the atrial tachycardia. The main limitation of this and similar work is that we know that many episodes of AF and atrial tachycardia are self-limited and brief, and therefore the quantification of efficacy may be clearly overestimated.

In the ATTEST study [54], the efficacy of atrial overdrive pacing in 368 patients possessing a pacemaker was evaluated. The overdrive pacing algorithms terminated 54% of the episodes, although the reduced percentage of atrial arrhythmias was not statistically significant: from 42 hours a month to 1.3 hours a month (p=0.2).

##  Conclusions

As we have seen in this review of the state-of-the-art, several parallel lines of research have been developed in recent years to treat patients with AF, either associated or not with bradycardia. The situation may seem confusing, and even more so if we consider some limitations of this work.

The first limitation is that in each study, we are analyzing something different that has not been studied previously. The data that we have from classical prevalence studies [1-3] are based on records, either with ECG or by Holter, of AF episodes, most of them symptomatic. In most of the mentioned studies, the presence or absence of AF is analyzed according to records of the pacemaker itself. This has two limitations. The first is that, as the devices and detection criteria are different, the data from a study may not be comparable to data from other studies. The second is that, as most AF episodes are asymptomatic, we are overestimating the incidence and it is not easy to extrapolate the data from these studies, which use symptomatic episodes as diagnostic or efficacy criteria.

Another limitation is that atrial fibrillation is a complex, dynamic arrhythmia. For reasons of protocol, the programming of devices in most of the trials has been based on a rigid, inflexible protocol [55]. Perhaps if there had been a little more freedom on adapting the algorithms to each patient, the degree of efficacy of the algorithms would have been higher. In addition, we are increasingly more aware of the adverse effect of VD apex pacing on the hemodynamics of these patients [55]. This effect at least partially explains the high incidence of atrial arrhythmias in patients with a permanent PM that was not accounted for in some studies. Perhaps in the future, with new algorithms tending to reduce VD pacing to the necessary minimum and with the expansion of resynchronization techniques, the approaches for treating atrial arrhythmias may yield greater benefits.

Finally, the follow-up of these studies has been very short. In Andersen's classical study [8], the AAI pacemaker did not yield benefits until after 5 years of follow-up; however, the data we have on these new techniques are limited to 6-month or one-year follow-ups. We need a longer follow-up to be able to accurately evaluate the efficacy of these devices.

From the data presented to date, we can conclude that the benefits obtained by implanting two electrodes, either in the right atrium or in both atria, are insignificant and probably do not justify the increased complexity of the implant. These techniques should be limited to research protocols, to try to identify the type of patient that would benefit the most.

The prevention algorithms are sound and barely raise the PM price, although their utility is limited. Nowadays, they could be used for different categories of patients:

1. Patients with PM indication and documented AF episodes.
2. Patients with a background of AF who develop secondary symptomatic bradycardia to medication.
3. Patients with paroxysmal atrial arrhythmias not suppressible by ablation and drug-refractory techniques for whom AV node ablation is considered as a therapeutic measure.

For the time being, the results of pacing do not justify its therapeutic use in patients with atrial fibrillation or atrial arrhythmias without associated bradycardia. One unanswered question is the optimum site of atrial pacing. Current data suggest a rather inconsistent benefit with septal pacing, whether it be from the coronary sinus ostium or Bachmann's bundle. But we still have very few data. Trials with more patients and longer follow-up periods are needed to change the current standard of implanting the atrial electrode in the appendage.

## Abbreviations

AF: Atrial Fibrillation
AE: Atrial Extra-systoles
SND: Sinus Node Dysfunction
PM: Pacemaker
PASE: Pacemaker Selection in the Elderly
CTOPP: Canadian Trial of Physiologic Pacing
MOST: Mode Selection Trial
UK-PACE: United Kingdom Pacing and Cardiovascular Events Trial
PA^3^: Atrial Pacing Periablation for Prevention of Paroxysmal Atrial Fibrillation:
AFT: Atrial Fibrillation Therapy Study
ASPECT: Atrial Septal Pacing Efficacy Clinical Trial
ATTEST: Atrial Therapy Efficacy and Safety Trial

## References

[R1] Kannel WB, Abbott RD, Savage DE (1982). Epidemiologic features of chronic atrial fibrillation: the Framingham Study. N Engl J Med.

[R2] Feinberg WM, Blackshear JL, Laupacis A (1995). Prevalence, age distribution, and gender of patients with atrial fibrillation: analysis and implications. Arch Intern Med.

[R3] Benjamin BJ, Wolf PA, D'Agostino RB (1998). Impact of atrial fibrillation on the risk of death: the Framingham Heart Study. Circulation.

[R4] Coplen SE, Antman EM, Berlin JA (1990). Efficacy and safety of quinidine therapy for maintenance of sinus rhythm after cardioversion: a metaanalysis of randomized controlled trials. Circulation.

[R5] Falk RH (1992). Proarrhythmia in patients treated for atrial fibrillation or flutter. Ann Intern Med.

[R6] Cox JL, Schuessler RB, D'Agostino HJ (1991). The surgical treatment of atrial fibrillation. III. Development of a definitive surgical procedure. J Thorac Cardiovasc Surg.

[R7] Jais P, Haissaguerre M, Shah DC (1997). A focal source of atrial fibrillation treated by discrete radiofrequency ablation. Circulation.

[R8] Andersen HR, Nielsen JC, Thomsen PEB (1997). Long-term follow-up of patients from a randomized trial of atrial versus ventricular pacing for sick sinus syndrome. Lancet.

[R9] Skanes AC, Krahn A, Yee R (2001). Progression to chronic atrial fibrillation after pacing: the Canadian trial of physiologic pacing. CTOPP investigators. J Am Coll Cardiol.

[R10] Lamas GA, Lee K, Sweeney M, for the MOST Investigators (2000). The mode selection trial (MOST) in sinus node dysfunction: Design, rationale, and baseline characteristics of the first 1000 patients. Am Heart J.

[R11] Daubert C, Mabo P, Berder V (1994). Atrial tachyarrhythmias associated with high degree interatrial conduction block: Prevention by permanent atrial resynchronization. Europace.

[R12] Saksena S, Prakash A, Hill M (1996). Prevention of recurrent atrial fibrillation with chronic dual site right atrial pacing. J Am Coll Cardiol.

[R13] Wijfells MC, Kirchhof CJ, Dorland RD (1995). Atrial fibrillation begets atrial fibrillation. A study in awake chronically instrumented goats. Circulation.

[R14] Saksena S, Prakash A, Krol RB (2000). Organized and reproducible atrial activation is present globally and regionally during phases of human atrial fibrillation [abstract]. J Am Coll Cardiol.

[R15] Coumel P, Friocourt P, Mugica J (1983). Long-term prevention of vagal atrial arrhythmias by atrial pacing at 90/minute: experience with 6 cases. PACE.

[R16] Attuel P, Pellerin D, Mugica J (1988). DDD pacing: an effective treatment modality for recurrent atrial arrhythmias. PACE.

[R17] Fitts SM, Hill MR, Mehra R (1998). Design and implementation of the dual site atrial pacing to prevent atrial fibrillation (DAPPAF) clinical trial. DAPPAF Phase 1 Investigators. J Intervent Card Electrophysiol.

[R18] Bailin SJ, Giudici MC, Solinger B (2001). Pacing from Bachmann's bundle prevents chronic atrial fibrillation: Final results from a prospective randomized trial [abstract]. PACE.

[R19] Prakash A, Saksena S, Hill M (1997). Acute effects of dual-site right atrial pacing in patients with spontaneous and inducible atrial flutter and fibrillation. J Am Coll Cardiol.

[R20] Murgatroyd FD, Nitzsche R, Slade AKB (1994). A new pacing algorithm for overdrive suppression of atrial fibrillation. PACE.

[R21] Prakash A, Saksena S, Ziegler P, for the DAPPAF Investigators (2001). Dual Site Atrial Pacing for Prevention of Atrial Fibrillation [DAPPAF] trial: echocardiographic evaluation of atrial and ventricular function during a randomized trial of support, high right atrial and dual site right atrial pacing [abstract]. PACE.

[R22] Israel CW, Huegl B, Unterberg-Buchwald C (2001). Performance of a new implantable DDRP device incorporating preventive and antitachycardia pacing modalities: Results of the international prospective AT500 verification study. PACE.

[R23] Andersen HR, Thuesen L, Bagger JP (1994). Thomsen PE: Prospective randomized trial of atrial versus ventricular pacing in sick sinus syndrome. Lancet.

[R24] Nielsen JC, Andersen HR, Thomsen PE (1998). Heart failure and echocardiographic changes during long-term follow-up of patients with sick sinus syndrome randomized to single-chamber atrial or ventricular pacing. Circulation.

[R25] Andersen HR, Nielsen JC, Thomsen PE (1999). Arterial thromboembolism in patients with sick sinus syndrome: Prediction from pacing mode, atrial fibrillation, and echocardiographic findings.. Heart.

[R26] Lamas GA, Orav EJ, Stambler BS (1998). Quality of life and clinical outcomes in elderly patients treated with ventricular pacing as compared to with dual-chamber pacing. Pacemaker Selection in the Elderly Investigators. N Engl J Med.

[R27] Mattioli AV, Vivoli D, Mattioli G (1998). Influence of pacing modalities on the incidence of atrial fibrillation in patients without prior atrial fibrillation. A prospective study. Eur Heart J.

[R28] Connolly SJ, Kerr CR, Gent M (2000). Effects of physiologic pacing versus ventricular pacing on the risk of stroke and death due to cardiovascular causes. Canadian Trial of Physiologic Pacing Investigators. N Engl J Med.

[R29] Tang AS, Roberts RS, Kerr C (2001). Relationship Between Pacemaker Dependency and the Effect of Pacing Mode on Cardiovascular Outcomes. Circulation.

[R30] Lamas GA, Lee KL, Sweeney MO, for Mode Selection Trial in Sinus-Node Function (2002). Ventricular pacing or dual-chamber pacing for sinus-node dysfunction. N Engl J Med.

[R31] Toff W, Skehan D (2003). Late Breaking Clinical Trials. oral presentation at the American College of Cardiology.

[R32] Gillis AM, Wyse DG, Connolly SJ (1999). Atrial pacing periablation for prevention of paroxysmal atrial fibrillation. Circulation.

[R33] Gillis AM, Connolly SJ, Lacombe P (2000). Randomized crossover comparison of DDDR versus VDD pacing after atrioventricular junction ablation for prevention of atrial fibrillation. Circulation.

[R34] Bayes de Luna A, Cladellas M, Oter R (1988). Interatrial conduction block and retrograde activation of the left atrium in paroxysmal supraventricular tachyarrhythmia. Eur Heart J.

[R35] Daubert JC, Leclercq C, Pavin D, Daubert JC, Prystowsky EN, Ripart A (1997). Biatrial synchronous pacing: A new approach to prevent arrhythmias in patients with atrial conduction block. Prevention of Tachyarrhythmias with Cardiac Pacing.

[R36] Pavin D, Revault D'Allonnes G, Mabo P, Israel  CW, Barold SS (2002). Left atrial and biatrial pacing. Advances in the Treatment of Atrial Tachyarrhythmias: Pacing, Cardioversion and Defibrillation.

[R37] Revault d'Allonnes G, Pavin D, Leclercq C (2000). Long-term effects of biatrial synchronous pacing to prevent drug-refractory atrial tachyarrhythmia: A nine-year experience. J Cardiovasc Electrophysiol.

[R38] Levy T, Fotopoulos G, Walker S (2000). Randomized controlled study investigating the effect of biatrial pacing in prevention of atrial fibrillation after coronary artery bypass grafting. Circulation.

[R39] Daoud EG, Dabir R, Archambeau M (2000). Randomized, double-blind trial of simultaneous right and left atrial epicardial pacing for prevention of post-open heart surgery atrial fibrillation. Circulation.

[R40] Fan K, Lee KL, Chiu CSW (2000). Effects of biatrial pacing in prevention of postoperative atrial fibrillation after coronary artery bypass surgery. Circulation.

[R41] Saksena S, Prakash A, Ziegler P (2002). Improved suppression of recurrent atrial fibrillation with dual-site right atrial pacing and antiarrhythmic drug therapy. J Am Coll Cardiol.

[R42] Padeletti L, Porciani MC, Michelucci A (1999). Interatrial septum pacing: A new approach to prevent recurrent atrial fibrillation. J Interv Card Electrophysiol.

[R43] Padeletti L, Pieragnoli P, Ciapetti C (2001). Randomized crossover comparison of right atrial appendage pacing versus interatrial septum pacing for prevention of paroxysmal atrial fibrillation in patients with sinus bradycardia. Am Heart J.

[R44] Kale M, Bennett DH (2002). Atrial septal pacing in the prevention of paroxysmal atrial fibrillation refractory to antiarrhythmic drugs. Int J Cardiol.

[R45] Bailin SJ, Adler S, Giudici M (2001). Prevention of chronic atrial fibrillation by pacing in the region of Bachmann's bundle: Results of a multicenter randomized trial. J Cardiovasc Electrophysiol.

[R46] Israel CW, Barold SS, Israel  CW, Barold SS (2002). Left atrial and biatrial pacing. Pacing algorithms for prevention of atrial tachyarrhythmias.

[R47] Carlson M, Ip J, Messenger J (2003). A New Pacemaker Algorithm for the Treatment of Atrial Fibrillation. Results of the Atrial Dynamic Overdrive Pacing Trial (ADOPT). J Am Coll Cardiol.

[R48] Camm AJ (2001). Preliminary results of the AF Therapy study. Presented at the Hotline Session of the XXIIII. Congress of the European Society of Cardiology, Stockholm.

[R49] Camm AJ (2002). AF therapy study: Preventive pacing for paroxysmal atrial fibrillation. (Abstract). Pacing Clin Electrophysiol.

[R50] Padeletti L, Purerfellner H, Adler S (2003). Combined Efficacy of Atrial Septal Lead Placement and Atrial Pacing Algorithms for Prevention of Paroxysmal Atrial Tachyarrhythmia. J Cardiovasc Electrophysiol.

[R51] Lozano IF, Vincent A, Roda J (2004). Paroxysmal atrial fibrillation prevention by pacing in patients with pacemaker indication. Europace.

[R52] Gregoratos G, Cheitlin MD, Conill A (1998). ACC/AHA Guidelines for Implantation of Cardiac Pacemakers and Antiarrhythmia Devices. Circulation.

[R53] Adler S, Wolpert C, Warman EN Efficacy of Pacing Therapies for Treating Atrial Tachyarrhythmias in Patients With Ventricular Arrhythmias Receiving a Dual-Chamber Implantable Cardioverter Defibrillator. Circulation.

[R54] Lee MA, Weachter R, Pollak S (2003). The Effect of Atrial Pacing Therapies on Atrial Tachyarrhythmia Burden and Frequency. J Am Coll Cardiol.

[R55] Wilkoff BL, Cook JR, Epstein AE (2002). Dual-chamber pacing or ventricular backup pacing in patients with an implantable defibrillator: The Dual-Chamber and VVI Implantable Defibrillator (DAVID) Trial. JAMA.

